# A Head-To-Head Comparison of Benzbromarone and Allopurinol on the Risk of Type 2 Diabetes Mellitus in People With Asymptomatic Hyperuricemia

**DOI:** 10.3389/fphar.2021.731370

**Published:** 2021-09-30

**Authors:** Shih-Wei Lai, Kuan-Fu Liao, Yu-Hung Kuo, Cheng-Li Lin, Chiu-Shong Liu, Bing-Fang Hwang

**Affiliations:** ^1^ Department of Public Health, College of Public Health, and School of Medicine, College of Medicine, China Medical University, Taichung, Taiwan; ^2^ Department of Family Medicine China Medical University Hospital, Taichung, Taiwan; ^3^ College of Medicine, Tzu Chi University, Hualien, Taiwan; ^4^ Division of Hepatogastroenterology, Department of Internal Medicine, Taichung Tzu Chi Hospita, Taichung, Taiwan; ^5^ Department of Research, Taichung Tzu Chi Hospita, Taichung, Taiwan; ^6^ School of Medicine, College of Medicine, China Medical University, Taichung, Taiwan; ^7^ Management Office for Health Data, China Medical University Hospital, Taichung, Taiwan; ^8^ Department of Occupational Safety and Health, College of Public Health, China Medical University, Taichung, Taiwan

**Keywords:** allopurinol, asymptomatic hyperuricemia, benzbromarone, cohort study, type 2 diabetes mellitus

## Abstract

**Objective:** The study aimed to thoroughly address the influence of benzbromarone and allopurinol on the risk of the development of type 2 diabetes mellitus (T2DM) in people with asymptomatic hyperuricemia.

**Methods:** We conducted a retrospective cohort study to examine the 2000–2015 national dataset containing all claims data of 23 million beneficiaries in Taiwan. Subjects who already had diabetes mellitus, gout-related diseases, and any cancer prior to the index date were excluded. Asymptomatic hyperuricemia was defined as subjects taking urate-lowering drugs who never had a gout flare. Subjects aged 20–84 with asymptomatic hyperuricemia who had benzbromarone prescriptions were selected as the benzbromarone group. Sex-matched and age-matched subjects with asymptomatic hyperuricemia who had allopurinol prescriptions were identified as the allopurinol group. The maximum follow-up duration was set as 5 years in our study. The outcome was set as subjects who had a new diagnosis of T2DM. The incidence density of T2DM was calculated in the benzbromarone and allopurinol groups. The hazard ratio (HR) and 95% confidence interval (CI) for T2DM was utilized to estimate the association between medications and the risk of T2DM.

**Results:** The incidence of T2DM among benzbromarone users was significantly lower than that of allopurinol users (7.91 versus 8.48 per 100 person-years, incidence rate ratio = 0.93, and 95% CI = 0.87–0.99). After adjustment for co-variables, the adjusted HR of T2DM would be 0.91 (95% CI = 0.85–0.98 and *p* = 0.008) in benzbromarone users as compared to allopurinol users.

**Conclusion:** There is a small but statistically significant risk reduction of developing T2DM in people with asymptomatic hyperuricemia taking benzbromarone as compared to those taking allopurinol during 5 years of follow-up. It indicates a future research direction for the use of individual urate-lowering drugs on the prevention of T2DM in the general population.

## Introduction

Diabetes mellitus (DM) is regarded as a global issue which has become a worldwide concern owing to its economic and medical burdens associated with diabetic complications. For example, DM-related healthcare expenditures were estimated to be 6.8 billion Euro in the Netherlands during 2016 (Peters et al., 2017), and 404 billion US dollars in USA during 2017 (Dall et al., 2019). In addition, DM ranked the fifth leading cause of death in Taiwan in 2020 (Ministry of Health and Welfare Taiwan, 2020). Especially type 2 diabetes mellitus (T2DM) accounted for more than 99% of the DM cases in Taiwan (Sheen et al., 2019).

Numerous factors and medical conditions have been detected to be related to an increased risk of developing T2DM. Some factors were non-modifiable including family history, older age, and genetics (Fletcher et al., 2002; Nguyen et al., 2015). Some factors were modifiable including hyperuricemia, dyslipidemia, hypertension, insulin resistance, obesity, decreased physical activity, and sedentary lifestyle (Fletcher et al., 2002; Nguyen et al., 2015; Bellou et al., 2018).

Hyperuricemia (higher serum level of uric acid) is regarded as a key factor for gout flares. Many studies have demonstrated a positive correlation of hyperuricemia with the risk ofT2DM. Moreover, one animal-model research has shown that the serum level of uric acid could be positively associated with insulin resistance in mice (Adachi et al., 2017). An observational study showed that after adjusting for diabetic risk factors, people with a higher level of serum uric acid might have a greater risk of developing T2DM as compared to people with lower serum level of uric acid (Sluijs et al., 2013). In addition, one 15-years follow-up cohort study in US showed that hyperuricemia was significantly related to a 1.87-fold greater risk of developing T2DM among people aged 18–30 (95%CI = 1.33–2.62) (Krishnan et al., 2012). Also, hyperuricemia was significantly related to 1.19-fold higher risk of T2DM development in the US veterans already having gout history (average age 62.9, 95%CI = 1.01–1.41) (Krishnan et al., 2013). But whether reducing the serum level of uric acid could decrease the probability of developing T2DM needs to be clarified. Recently observational studies have shown conflicting results in the association between urate-lowering drugs and the risk of T2DM. One study showed that gout people with urate-lowering drugs use were at greater risk of developing T2DM (Chang et al., 2019). To the contrary, other studies have shown that the use of urate-lowering drugs was significantly related to a decreased risk of developing T2DM (Niu et al., 2018; Fang et al., 2020).

To date, whether people with asymptomatic hyperuricemia need administering urate-lowering drugs remains to be determined. Also, no research examines the association between urate-lowering drugs and the probability of developing T2DM among people with asymptomatic hyperuricemia. If the relationship is illustrated, the treatment policy for asymptomatic hyperuricemia will be clear. Therefore, a head-to-head comparison was made to address the influence of benzbromarone and allopurinol on the risk of developing T2DM in people with asymptomatic hyperuricemia.

## Methods

### Research Design, Data Source, and Research Subjects

This study was a retrospective cohort design utilizing the 2000–2015 claims data of the National Health Insurance Program in Taiwan.

Asymptomatic hyperuricemia was defined as subjects taking urate-lowering drugs who never had a gout flare. The index date was set to the date when the uric acid-lowering drugs were prescribed. Subjects who had cumulative use duration of the urate-lowering drugs ≥30 days could be included in the study. Subjects aged 20–84 with asymptomatic hyperuricemia who ever had a history of benzbromarone prescriptions were selected as the benzbromarone group. Subjects aged 20–84 with asymptomatic hyperuricemia who ever had a history of allopurinol prescriptions were selected as the allopurinol group. The two groups of benzbromarone and allopurinol were matched by sex, age, studied medications, studied comorbidities, as well as the index year. The comorbidities were defined based on International Classification of Diseases 9th Revision Clinical Modification (ICD-9 codes).

**FIGURE 1:**
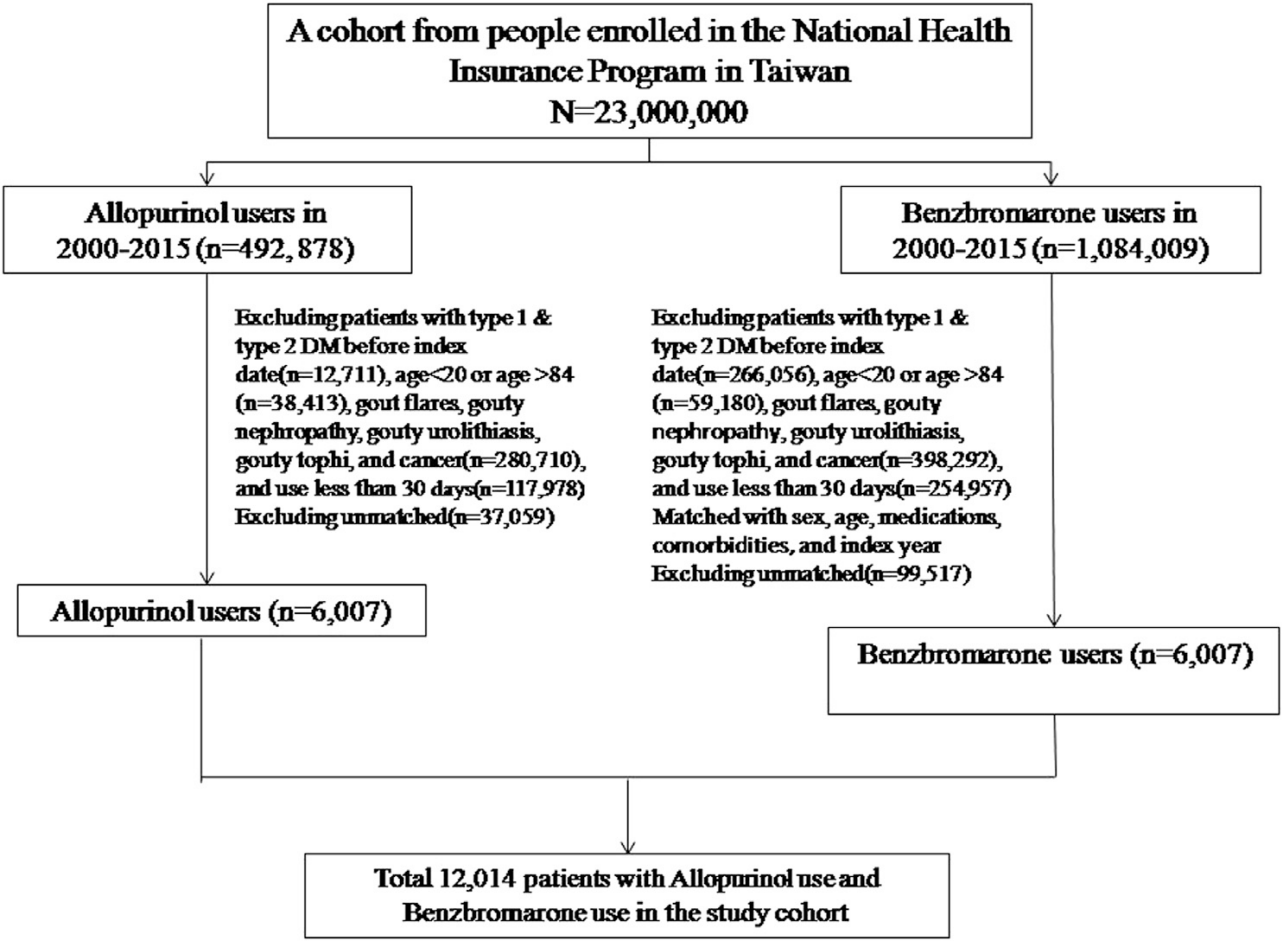
Flowchart showing selection process of study subjects.

The following subjects were excluded from the study: 1) subjects who already had been diagnosed with diabetes mellitus prior to the index date, 2) subjects who had suffered from gout-related diseases (including joint gout flares, gouty nephropathy, gouty urolithiasis, and gouty tophi) before the index date and during the cohort period, 3) subjects who had suffered from any cancer before the index date and during the cohort period, 4) subjects who had co-administration of any two urate-lowering drugs during the cohort period (Figure 1).

### Main Outcome

The maximum follow-up duration was set as 5 years in our study. The main outcome was defined as subjects who had a new diagnosis of T2DM (based on International Classification of Diseases 9^th^ Revision Clinical Modification, ICD-9 codes 250. x0 or 250. x2 with x = 0–9). Only those subjects who had the same diagnosis of T2DM ≥ 3 records in outpatient department and/or ≥1 record in hospitalization during the cohort could be included for analysis. Previous research reported the validity of the DM diagnosis based-on ICD-9 codes in Taiwan, with a positive predictive value of 90% and a negative predictive value of 97% (Cheng et al., 2011).

### Statistical Analyses

We applied a propensity score matching based on sex, age, studied medications, and studied comorbidities listed on Table 1. The number and percentage were used in the exhibition of the categorical variables. The mean with standard deviation was used in the exhibition of the continuous variables. The comparison analysis was performed by utilizing the Chi-square test between the categorical variables and using the *t*-test between the continuous variables. In addition, the incidence density of T2DM was measured in person-years in the benzbromarone and allopurinol groups. The cumulative incidence of T2DM was revealed by Kaplan-Meier curve, and the difference was presented by log-rank test. The proportional hazard assumption was examined using a test of scaled Schoenfeld residuals. In the model evaluating the risk of T2DM throughout overall follow-up time, the results of the scaled Schoenfeld residuals test revealed a significant relationship for medication exposure and follow-up time (*p* value = 0.001), which suggested the proportionality assumption was violated. In the subsequent analyses, we stratified the follow-up time to deal with the violation of proportional hazard assumption. The hazard ratio (HR) with 95% confidence interval (CI) was utilized to estimate the association between sex, age, studied medications, studied comorbidities, and the risk of T2DM by the Cox proportional hazard regression model. The cumulative doses of benzbromarone use were measured to explore the risk of the development of T2DM.The SAS 9.4-version software was applied to carry out the statistic analysis. The *p* value <0.05 would be significant.

**TABLE 1:** Baseline information of study subjects after propensity score matching.

	Benzbromarone N = 6,007		Allopurinol N = 6,007		
Variable	n	(%)	n	(%)	*p* value^a^
Sex					0.56
Male	4,422	73.6	4,450	74.1	
Female	1,585	26.4	1,557	25.9	
Age (years)					0.70
20–39	991	16.5	997	16.6	
40–64	2,910	48.4	2,865	47.7	
65–84	2,106	35.1	2,145	35.7	
Mean ± SD^b^	57.0 ± 16.2		57.1 ± 16.3		0.81
Medications					
Ever use of thiazide diuretics	2,196	36.6	2,246	37.4	0.34
Ever use of loop diuretics	1701	28.3	4,680	28.0	0.67
Ever use of aspirin	2,226	37.1	2,292	38.2	0.21
Baseline comorbidities					
Cerebrovascular disease	492	8.19	485	8.07	0.82
Chronic kidney disease	1,135	18.9	1,146	19.1	0.80
Chronic obstructive pulmonary disease	1,151	19.2	1,182	19.7	0.48
Coronary artery disease	1,646	27.4	1,680	28.0	0.49
Hyperlipidemia	2087	34.7	2083	34.7	0.94
Hypertension	3,757	62.5	3,758	62.6	0.99

Data are presented as the number of subjects in each group, with percentages given in parentheses.

SD: standard deviation.

a Chi-square test.

b *t*-test comparing the benzbromarone group and the allopurinol group.

## Results

### Baseline Information of the Study Population

Totally, 6,007 eligible subjects in the benzbromarone group and 6,007 eligible subjects in the allopurinol group were identified after propensity score matching (Table 1). The distribution of sex and age was similar in the benzbromarone and the allopurinol groups. The male subjects were predominant (about 74%). The average age in both groups was 57 years old. No statistical difference in the use of studied medications and comorbidities was detected between these two groups (Chi-square test, *p* > 0.05).

Among 92.1% of benzbromarone users, the total duration of benzbromarone use was ≤540 days. The rest of 7.9% was >540 days. Among 89.7% of allopurinol users, the total duration of allopurinol use was ≤540 days. The rest of 10.3% was >540 days (Table not shown).

### Incidence Density of Type 2 Diabetes Mellitus Stratified by Subject Characteristics

The overall incidence rate of T2DM among benzbromarone users was significantly lower than that of allopurinol users (7.91 versus 8.48 per 100 person-years, incidence rate ratio = 0.93, 95% CI = 0.87–0.99, and *p* = 0.031). In Table 2, the incidence rate of T2DM among benzbromarone users was lower than that of allopurinol users during the initial 3.5 years of follow-up (5.08 versus 5.74 per 100 person-years, adjusted HR = 0.87, 95% CI = 0.79–0.95, and *p* = 0.002). Even after 3.5 years of follow-up, the incidence rate of T2DM among benzbromarone users was still lower than that of allopurinol users, although not reaching statistical significance (27.3 versus 28.0 per 100 person-years, adjusted HR = 0.98, 95% CI = 0.88–1.09, and *p* = 0.70).

**TABLE 2:** Incidence density of type 2 diabetes mellitus between benzbromarone use group and allopurinol use group.

	Benzbromarone				Allopurinol					
Variable	N	Event	Person-years	Incidence rate	N	Event	Person-years	Incidence rate	Crude HR^#^ (95% CI) (*p* value)	Adjusted HR^#^ (95% CI) (*p* value)
Follow-up duration (years)										
<3.5	6,007	941	18,530	5.08	6,007	1,022	17,791	5.74	0.88 (0.80, 0.96) (0.004)	0.87 (0.79, 0.95) (0.002)
3.5–5	5,693	738	2,700	27.3	5,524	700	2,504	28.0	0.98 (0.89, 1.09) (0.72)	0.98 (0.88, 1.09) (0.70)

Incidence rate: 100 person-years; HR: Hazard ratio; 95%CI: 95% confidence interval. ^#^By the Cox proportional hazard regression model, adjusted for sex, age, thiazide diuretics use, loop diuretics use, aspirin use, cerebrovascular disease, chronic kidney disease, chronic obstructive pulmonary disease, coronary artery disease, hyperlipidemia and hypertension

**FIGURE 2:**
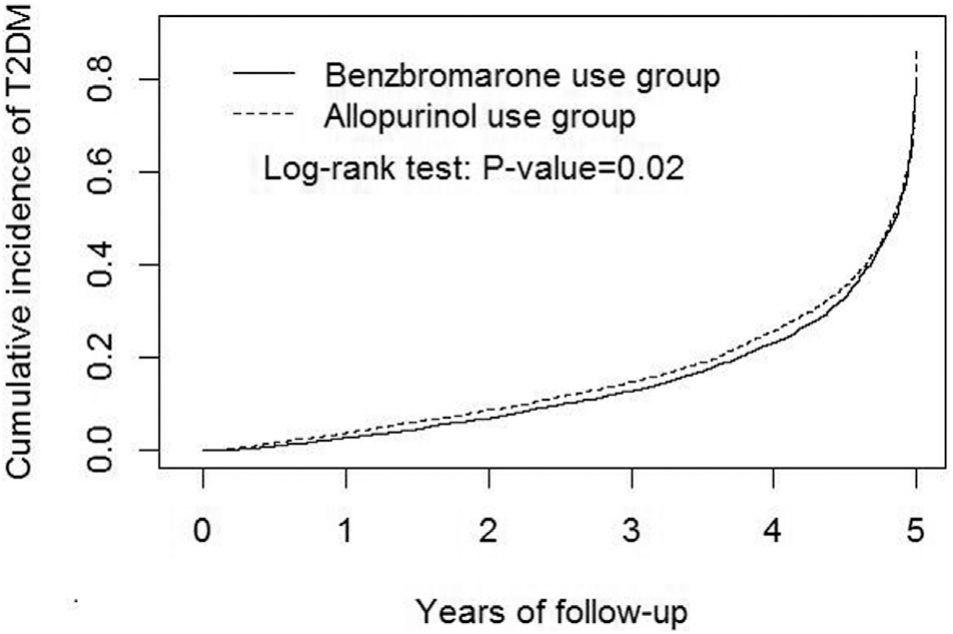
Kaplan-Meier model demonstrated that the cumulative incidence of type 2 diabetes mellitus was lower in benzbromarone users than that in allopurinol users at the end of the follow-up period (*p* = 0.02).

In Figure 2, the Kaplan-Meier model demonstrated that the cumulative incidence of T2DM among benzbromarone users was significantly lower than that of allopurinol users at the end of the follow-up period (*p* = 0.02).

In Table 3, the mean follow-up time among benzbromarone users was significantly longer than that of allopurinol users (mean ± SD, 3.53 ± 1.12 versus 3.38 ± 1.25 years, *p* < 0.001). The mean duration from the index date to the development of new-onset T2DM among benzbromarone users was significantly longer than that of allopurinol users (mean ± SD, 3.04 ± 1.33 versus 2.88 ± 1.38 years, *p* = 0.001).

**TABLE 3:** Mean follow-up time and mean duration from the index date to new-onset type 2 diabetes mellitus (T2DM).

Variable	Benzbromarone	Allopurinol	*p* Value
Mean follow-up time (SD, years)	3.53 (1.12)	3.38 (1.25)	<0.001
Mean duration from the index date to new-onset T2DM (SD, years)	3.04 (1.33)	2.88 (1.38)	0.001

SD: standard deviation.

### Hazard of Type 2 Diabetes Mellitus Related to Medications and Comorbidities

In Table 4, after adjustments for co-variables, the multivariable Cox proportional hazard regression model demonstrated that benzbromarone users had a lower hazard of T2DM than allopurinol users (adjusted HR = 0.91, 95%CI = 0.85–0.98, and *p* = 0.008).

**TABLE 4:** Hazard ratio and 95% confidence interval of type 2 diabetes associated with medications and comorbidities by the Cox proportional hazard regression model.

Variable	Crude			Adjusted^a^		
	HR	(95%CI)	*p* Value	HR	(95%CI)	*p* Value
Sex (female vs. male)	1.60	(1.49, 1.72)	<0.001	1.35	(1.25, 1.45)	<0.001
Age (every 1 year)	1.02	(1.02, 1.03)	<0.001	1.02	(1.02, 1.02)	<0.001
Benzbromarone use (allopurinol use as a reference)	0.92	(0.86, 0.98)	0.015	0.91	(0.85, 0.98)	0.008
Medications						
Thiazide diuretics use (non-use as a reference)	1.29	(1.21, 1.39)	<0.001	0.87	(0.81, 0.95)	<0.001
Loop diuretics use (non-use as a reference)	1.25	(1.16, 1.34)	<0.001	0.95	(0.88, 1.04)	0.26
Aspirin use (non-use as a reference)	1.18	(1.11, 1.27)	<0.001	0.81	(0.74, 0.88)	<0.001
Baseline comorbidities (yes vs. no)						
Cerebrovascular disease	1.50	(1.34, 1.68)	<0.001	1.16	(1.03, 1.31)	0.02
Chronic kidney disease	0.92	(0.84, 1.01)	0.08			
Chronic obstructive pulmonary disease	1.31	(1.20, 1.42)	<0.001	1.00	(0.91, 1.09)	0.99
Coronary artery disease	1.54	(1.43, 1.65)	<0.001	1.25	(1.14, 1.36)	<0.001
Hyperlipidemia	1.06	(0.99, 1.14)	0.10			
Hypertension	1.76	(1.63, 1.89)	<0.001	1.44	(1.31, 1.58)	<0.001

a Adjusted for sex, age, thiazide diuretics use, loop diuretics use, aspirin use, cerebrovascular disease, chronic obstructive pulmonary disease, coronary artery disease, and hypertension.

### Hazard of Type 2 Diabetes Mellitus Related to Cumulative Doses of Benzbromarone Use

In Table 5, with Q1 cumulative doses of benzbromarone use as a reference, the adjusted HR of T2DM was 0.89 in the group of Q2 cumulative doses (95% CI = 0.78–1.02 and *p* = 0.10). The adjusted HR of T2DM was 0.91 in the group of Q3 cumulative doses (95% CI = 0.79–1.04 and *p* = 0.16). The adjusted HR of T2DM was 0.78 in the group of Q4 cumulative doses (95% CI = 0.68–0.90 and *p* < 0.001).

**TABLE 5:** Hazard ratio and 95% confidence interval of type 2 diabetes associated with cumulative doses of benzbromarone use.

Variable	Crude			Adjusted^a^		
	HR	(95%CI)	*p* Value	HR	(95%CI)	*p* Value
Q1 cumulative MG as a reference (N = 1,518)^b^						
Q2 cumulative MG (N = 1,493)	0.88	(0.76, 1.00)	0.05	0.89	(0.78, 1.02)	0.10
Q3 cumulative MG (N = 1,536)	0.89	(0.78, 1.02)	0.09	0.91	(0.79, 1.04)	0.16
Q4 cumulative MG (N = 1,460)	0.80	(0.70, 0.91)	0.001	0.78	(0.68, 0.90)	<0.001

a Adjusted for sex, age, thiazide diuretics use, loop diuretics use, aspirin use, cerebrovascular disease, chronic obstructive pulmonary disease, coronary artery disease, and hypertension.

b Cumulative MG: cumulative dose (mg).

## Discussion

We observed that people with asymptomatic hyperuricemia taking benzbromarone had 9% risk reduction of developing T2DM when compared to those taking allopurinol after adjustments for co-variables (Table 4). There seemed to be a dose-dependent relationship between the cumulative doses of benzbromarone use and the risk reduction of T2DM development (Table 5). We observed that the mean duration from the index date to the development of new-onset T2DM among benzbromarone users was significantly longer than that of allopurinol users (Table 2). It means that benzbromarone use may delay the onset of T2DM.

Epidemiological studies have reported that people with hyperuricemia had a greater risk of T2DM development in the future (Krishnan et al., 2012; Krishnan et al., 2013; Sluijs et al., 2013). We observed that the overall incidence density of T2DM would be 8.19 per 100 person-years among people with asymptomatic hyperuricemia in our study. An observational study in Taiwan showed that the incidence density of DM in the general population ages 20–79 years would be 11.9 per 10,000 persons in 2005 (Sheen et al., 2019). It means that the probability of developing T2DM in people with asymptomatic hyperuricemia was about 69 times greater than that of the general population. More efforts should be intervened to diminish the risk of developing T2DM in people with asymptomatic hyperuricemia.

The relationship between the reduction of the serum level of uric acid and the probability of T2DM development needs to be tested. One study by Niu et al. disclosed that gout people with benzbromarone use were related to a lower risk of DM development as comparing to those people with non-use of benzbromarone (HR = 0.85; 95%CI = 0.77–0.93) (Niu et al., 2018). But Niu et al.’s study did not mention that non-use of benzbromarone was those taking other urate-lowering drugs or those with no use of any urate-lowering drugs. One study by Chang et al. demonstrated that gout people with urate-lowering drugs use were related to a greater risk of developing T2DM as compared to those people with no gout (HR = 1.09, 95% CI = 1.03–1.15 for benzbromarone and HR = 1.17, 95% CI = 1.07–1.28 for allopurinol, respectively) (Chang et al., 2019). One study by Fang et al. demonstrated that gout people with urate-lowering drugs use were related to a lower risk of T2DM development as comparing to gout people with no use of anti-gout treatment (HR = 0.89, 95%CI = 0.86–0.93 for benzbromarone and HR = 0.57, 95% CI = 0.54–0.61 for allopurinol, respectively) (Fang et al., 2020). Based on a symmetric comparison, the therapeutic group should include hyperuricemic people with urate-lowering drugs use. Theoretically, the comparison group should include hyperuricemic people with no use of urate-lowering drugs. In the above two studies (Chang et al., 2019; Fang et al., 2020), the therapeutic group included gout people with urate-lowering drugs use. It is a reasonable inclusion. But Chang et al.’s study only included people with no gout history as the comparison group. Such a comparison group might have a selection bias because people with no gout history might have asymptomatic hyperuricemia or normal serum level of uric acid. That was why there were conflicting results in the above two studies, which could be due to the selection bias presented in the comparison group. It should be cautious when interpreting their results.

We found that benzbromarone and allopurinol accounted for approximately 92% of the prescriptions of urate-lowering drugs in Taiwan in a preliminary analysis. We decided to directly compare the efficacy of benzbromarone and allopurinol. The causal relationship between hyperuricemia andT2DM remains unclear. The mechanism underlying their link is not the scope of our study. We reviewed the literature and summarized that a high level of serum uric acid might induce pancreatic beta-cell dysfunction and later developing T2DM (Ghasemi, 2021). The vitro study showed that uric acid could enter the pancreatic beta-cells via glucose transporter 9 (GLUT9) (Evans et al., 2009). Thus, the high level of intracellular uric acid within the pancreatic beta-cells might lead to the overproduction of nitric oxide (NO), which causes inflammation of the pancreatic beta-cells, the decrease of glucose-stimulated insulin secretion, and also causes apoptosis of the pancreatic beta-cells (Rocić et al., 2005; Ghasemi, 2021). In addition, the high level of intracellular uric acid within the pancreatic beta-cells might lead to the increase of reactive oxygen species (ROS), which also causes apoptosis of pancreatic beta-cells (Ghasemi, 2021). Urate-lowering drugs could lower the level of uric acid and theoretically might protect the pancreatic beta-cells against attacks from nitric oxide and reactive oxygen species. Consequently, urate-lowering drugs might be beneficial for the prevention of T2DM.

Uric acid elimination is mainly regulated by the kidneys and the gut in humans (Dalbeth et al., 2016; Mortada, 2017). The kidneys account for about 70% of elimination of the daily uric acid production (Maesaka and Fishbane, 1998; Anzai et al., 2005). Renal transporters play the dominant role in the regulation of the reabsorption of urinary uric acid. Variants of these renal transporters are noted to be related to the development of hyperuricemia and gout. Urate transporter 1 (URAT1, encoded by SLC22A12 gene), being a transporter protein, is localized to the apical membrane of the renal proximal tubular cells (Enomoto et al., 2002; Anzai et al., 2005). URAT1 has a major function on the reabsorption of urinary uric acid (Enomoto et al., 2002; Anzai et al., 2005). Moreover, URAT1 is the key renal transporter responsible for the regulation of the serum level of uric acid (Anzai et al., 2005). Theoretically, inhibiting URAT1 by pharmacological compounds can increase the excretion of urinary uric acid, can lower the serum level of uric acid, and therefore can prevent gout flares. Benzbromarone is a high-affinity inhibitor of URAT1 and it is also a potent uricosuric drug clinically (Anzai et al., 2005). Sodium-glucose co-transporter 2 (SGLT2, encoded by SLC5A2 gene)*,* which is a sodium-glucose co-transporter protein, is localized to the apical membrane of the renal proximal tubular cells (Gerich, 2010; Ghezzi et al., 2018). SGLT2 has a major role on the reabsorption of urinary glucose (Gerich, 2010; Ghezzi et al., 2018). Theoretically, inhibiting SGLT2 by pharmacological compounds can increase the excretion of urinary glucose and consequently can reduce the serum level of glucose in patients with T2DM (Schwartz and Ahmed, 2016; Derosa and Maffioli, 2018). URAT1 itself is not a glucose transporter, but URAT1 and SGLT2 might interact with each other to influence their functions. We propose a hypothesis that benzbromarone not only is a URAT1 inhibitor with a potent uricosuric effect, but also may have a partial inhibiting effect on SGLT2. Such an inhibiting effect on SGLT2 could increase the excretion of urinary glucose and then could reduce the serum level of glucose. Thus, the probability of developingT2DM could be further reduced.

Allopurinol, one xanthine oxidase inhibitor, is widely prescribed to treat people with overproduction-type hyperuricemia (Benn et al., 2018; Strilchuk et al., 2019). Allopurinol can lower the level of uric acid and theoretically might protect the pancreatic beta-cells against attacks from nitric oxide and reactive oxygen species. A prospective cohort study showed that allopurinol use could improve insulin resistance in asymptomatic hyperuricemic non-diabetic people (Takir et al., 2015). To the contrary, a retrospective cohort study showed that allopurinol use was not related to a decreased risk of T2DM (Slobodnick et al., 2020). The role of allopurinol on the risk of the development of T2DM remained unsettled. More studies are required to explore the impact of allopurinol on the development risk of T2DM.

Medication safety needs to be discussed. Due to its potential hepatotoxicity, benzbromarone has exited from some European markets since 2003 (Jansen et al., 2004). Because the benzbromarone-related research was little, we were unable to cite for comparison. To date, no case was reported about benzbromarone-related serious side effects in Taiwan. That is why benzbromarone still exists in Taiwan even where there is a high prevalence of hepatitis B carriers. In addition, the incidence rate of allopurinol-related severe cutaneous adverse reactions was 2.57 per 1,000 person-years in Taiwan (Sato et al., 2021). Therefore, benzbromarone is relative safe than allopurinol in Taiwan.

### Limitation

First, the serum uric acid was not routinely checked in Taiwan. Whether the general population had a normal or high serum level of uric acid could not be differentiated from the claims data of the National Health Insurance Program in Taiwan. So it was difficult to select people with hyperuricemia who did not take urate-lowering drugs from the general population as comparisons. At present, we could only compare the relative risk of the development of T2DM between benzbromarone users and allopurinol users. Second, due to lack of the relevant data on the serum level of uric acid as baseline, during or post use of benzbromarone and allopurinol, body mass index, as well as health behaviors, rigorous confounding adjustment could not be performed in our study. Third, owing to lack of the lab data of blood sugar and hemoglobin A1c, we could not evaluate whether the baseline glycemic status would affect the risk of developing T2DM. Fourth, many factors including non-modifiable and modifiable, have been observed to be related to a greater risk of developing T2DM. As the time goes by, one of the main influencing factors for developing T2DM could be the time. It is less likely that the individual drug could have a main influence on the prevention of T2DM. That was why the incidence rates of T2DM among benzbromarone users and allopurinol users were close after 3.5 years of follow-up (27.3 versus 28.0 per 100 person-years). However, people with asymptomatic hyperuricemia can be advised on lifestyle changes such as changes in diet, regular exercise, and reduction of alcohol intake, all of which may reduce the risk of developing T2DM.Fifth, the outcome algorithm has not been validated in our study. However, only those subjects who had the same diagnosis of T2DM ≥ 3 records in outpatient department and/or ≥1 record in hospitalization during the cohort could be included for analysis. The possibility of T2DM misclassification bias could be minimal in our study. Sixth, due to the limitation of the database used, the indications for benzbromarone and allopurinol were not addressed. We could not control the confounding by the indication between benzbromarone and allopurinol. Seventh, among 92.1% of benzbromarone users and 89.7% of allopurinol users, the total duration of drug use was ≤540 days during the cohort period. So it was reasonable to set the follow-up duration as 5 years in our study.

## Conclusion

There is a small but statistically significant risk reduction of developing T2DM in people with asymptomatic hyperuricemia taking benzbromarone as compared to those taking allopurinol during 5 years of follow-up. Moreover, randomized controlled trials are needed to explore if the use of individual urate-lowering drugs can prevent the development of T2DM in the general population.

## Data Availability

The original contributions presented in the study are included in the article. Further inquiries can be directed to the corresponding author.
